# Optimizing Imaging Techniques for Left Atrial Appendage Closure: Insights and Emerging Directions

**DOI:** 10.3390/jcm14103607

**Published:** 2025-05-21

**Authors:** Valentina Barletta, Mattia Alberti, Riccardo Agostini, Fausto Pizzino, Giancarlo Trimarchi, Maria Grazia D’Alfonso, Marco Solari, Giulio Zucchelli, Alberto Cresti

**Affiliations:** 1 Department of Cardiac-Thoracic and Vascular, Second Division of Cardiology, Pisa University Hospital, 56100 Pisa, Italy; 2 Department of Surgical, Medical and Molecular Pathology and Critical Area, Cardiology Division, University of Pisa, 56126 Pisa, Italy; 3 Department of Cardiac, Thoracic, and Vascular Medicine, Azienda Ospedaliero Universitaria Careggi, 50134 Florence, Italy; 4 Fondazione Toscana G. Monasterio, Ospedale del Cuore, 54100 Massa, Italy; 5 Department of Cardiology, S. Giuseppe Hospital, 50053 Empoli, Italy; 6 Cardiology Department, Misericordia Hospital, Azienda Sanitaria Toscana SudEst, 58100 Grosseto, Italy

**Keywords:** left atrial appendage, oral anticoagulants, thrombus, multi-imaging, echocardiography

## Abstract

Atrial fibrillation (AF) is the most common sustained cardiac arrhythmia in adults and is associated with significant morbidity and mortality, including an increased risk of stroke, heart failure, dementia, and recurrent hospitalizations. As life expectancy rises, both the incidence and prevalence of AF continue to grow. Stroke prevention remains a cornerstone of AF management, with oral anticoagulation being the primary strategy to reduce thromboembolic risk. However, despite their advantages, direct oral anticoagulants do not completely eliminate the risk of bleeding complications. For patients in whom anticoagulation is contraindicated, poorly tolerated, or ineffective at preventing AF-related stroke, interventional alternatives have gained traction. The left atrial appendage (LAA), a primary site of thrombus formation in AF, can be occluded through a catheter-based procedure known as left atrial appendage closure (LAAC) or left atrial appendage occlusion (LAAO). This review aims to provide imaging specialists with a comprehensive understanding of their role in LAAC, underscoring the importance of a multidisciplinary approach to enhance patient selection, procedural success, and long-term efficacy.

## 1. Introduction

Atrial fibrillation (AF) is the most common sustained cardiac arrhythmia in adults and is associated with significant morbidity and mortality, including an increased risk of stroke, heart failure, dementia, and recurrent hospitalizations. As life expectancy rises, both the incidence and prevalence of AF continue to grow [1].

Stroke prevention remains a cornerstone of AF management, with oral anticoagulation being the primary strategy to reduce thromboembolic risk. In recent years, direct oral anticoagulants (DOACs) have largely replaced vitamin K antagonists (VKAs) for patients without significant valvular heart disease, particularly in Europe and North America, due to their favorable safety profile. However, despite their advantages, DOACs do not completely eliminate the risk of bleeding complications [2].

For patients in whom anticoagulation is contraindicated, poorly tolerated, or ineffective at preventing AF-related stroke, interventional alternatives have gained traction. The left atrial appendage (LAA), a primary site of thrombus formation in AF, can be occluded through a catheter-based procedure known as left atrial appendage closure (LAAC) or left atrial appendage occlusion (LAAO). Clinical trials and guideline recommendations have established LAAC as a valuable option for stroke prevention in high-risk patients while simultaneously reducing the long-term bleeding risks associated with anticoagulation therapy [3,4].

Although LAAC is primarily performed by interventional cardiologists, the procedure is heavily dependent on advanced imaging techniques. Accurate pre-procedural assessment, real-time intra-procedural guidance, and post-procedural follow-up are essential to optimize outcomes.

This review aims to provide imaging specialists with a comprehensive understanding of their role in LAAC, underscoring the importance of a multidisciplinary approach to enhance patient selection, procedural success, and long-term efficacy. In this narrative review, we conducted a structured literature search using PubMed for each key section of the manuscript. Boolean operators were used to construct search strings, such as (“left atrial appendage closure” OR “left atrial appendage occlusion”) AND (“transesophageal echocardiography”) AND (“cardiac CT”) AND (“fusion imaging”). Similar combinations were adapted for other imaging modalities and technologies covered. We prioritized studies based on relevance (“Best Match” sorting), recency, and citation frequency, with particular attention to major clinical guidelines and systematic reviews. Only articles published in English were considered. Studies unrelated to imaging in LAAC or lacking sufficient methodological detail were excluded.

The role of multimodality imaging in LAAC is increasingly supported by international guidelines. According to the 2021 ESC guidelines on cardiac pacing and cardiac resynchronization therapy [1] and the 2023 AHA/ACC guideline for the management of patients with atrial fibrillation, imaging is essential for patient selection, procedural guidance, and post-procedural assessment, with specific modalities recommended depending on the clinical context and institutional expertise (Class IIa–IIb recommendations). These recommendations highlight the growing importance of standardized imaging protocols in optimizing procedural success and minimizing complications (see Supplementary Materials for more details [1,5,6]). Pre-procedural planning often involves contrast-enhanced computed tomography (CECT) to assess LAA morphology and optimal device selection. During the procedure, transesophageal echocardiography (TEE) or intracardiac echocardiography (ICE) provide real-time visualization, guiding device deployment and confirming proper positioning. Fluoroscopy remains the primary imaging modality for device placement and, in selected cases, fusion imaging techniques integrating CECT or TEE with fluoroscopy may enhance procedural accuracy. Post-procedural imaging is crucial for detecting residual leaks, device-related thrombus, or other complications [7].

## 2. Role of Imaging in Left Atrial Appendage Closure

### 2.1. Pre-Procedural Imaging: Anatomical Assessment and Patient Selection

The LAA is a remnant of the primitive atrium, developing in the third gestational week. Its structure considerably varies among individuals, influencing procedural planning and device selection for LAAC [8].

The most widely used classification of LAA morphology, introduced by Wang et al., identifies four distinct types based on computed tomography (CT) imaging [9].

- Chicken Wing: A dominant lobe bending <100° in its proximal segment.
- Cactus: A central lobe <40 mm with secondary lobes and recesses.
- Windsock: A dominant lobe >40 mm with secondary lobes bending >100 mm.
- Cauliflower: A short, irregular lobe <40 mm with multiple recesses.

Understanding these morphological variations is crucial for selecting the most appropriate occlusion device as anatomical factors influence procedural success and complication risks. As highlighted by Cresti et al., key considerations include the ostium size; LAA depth and width; landing zone characteristics; proximity to the circumflex artery, pulmonary ridge, and mitral valve; and the presence of trabeculations or secondary lobes [10]. Optimal device selection requires precise measurements to ensure proper deployment while minimizing the risks of embolization, peri-device leak, and device-related thrombus.

**Figure 1:**
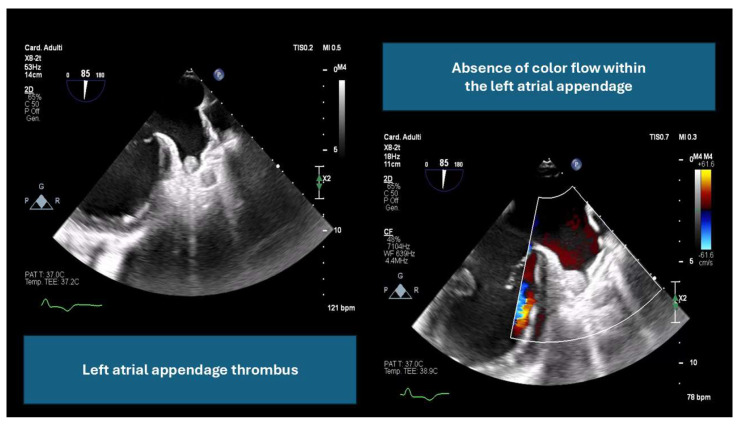
Transesophageal echocardiographic image showing evidence of left atrial appendage thrombus. A hyperechoic mass is visible, adherent to the wall of the left atrial appendage, consistent with thrombus.

Multimodality imaging plays a critical role in procedural planning [11,12] as follows:

1. By ruling out LAA thrombus, which can increase the risk of embolic events (an example is provided in Figure 1).
2. By providing detailed anatomical assessment, including LAA dimensions, peak emptying velocity, and surrounding structures.
3. By determining optimal fluoroscopic angles and guiding transseptal puncture locations for accurate device delivery.

**Figure 2:**
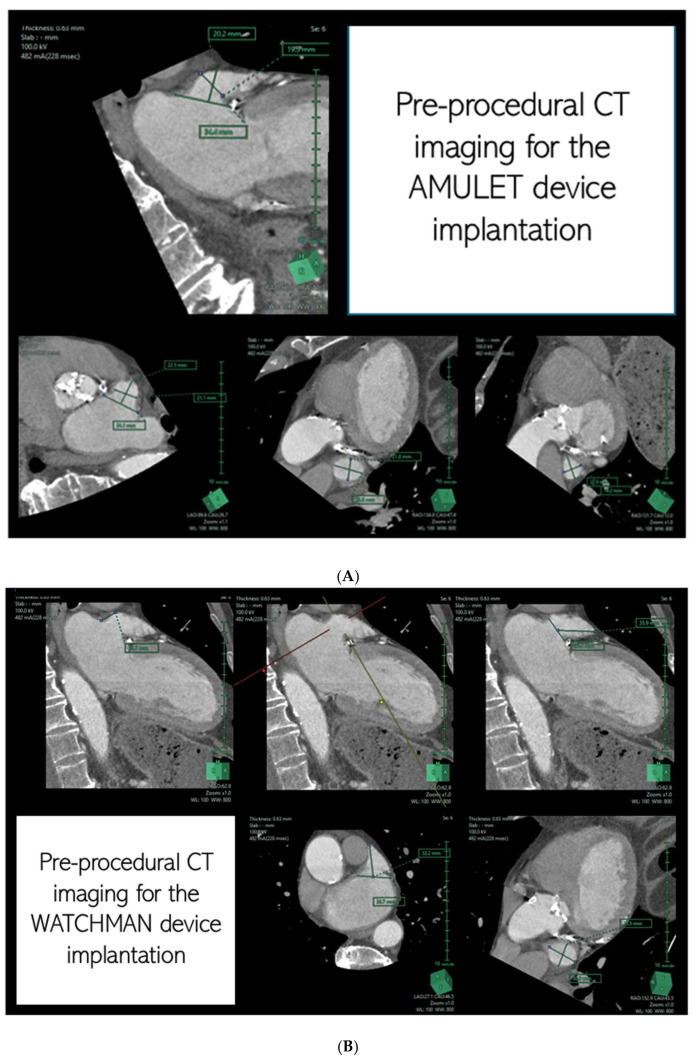
Contrast-enhanced computed tomography (CECT) with multiplanar reformation (MPR) of the left atrial appendage. The images represent the pre-procedural measurements essential for selecting the appropriate size for the AMULET (**A**) and WATCHMAN (**B**) closure devices.

CECT has become a preferred modality for LAA assessment due to its ability to generate high-resolution 3D volumetric images across the cardiac cycle. It provides detailed insights into LAA anatomy, facilitates device selection, and aids in the post-procedural evaluation of peri-device leaks. Additionally, its capacity to simulate fluoroscopic imaging angles allows operators to plan optimal C-arm positions, reducing procedural complexity [13] (Figure 2A,B).

Although TEE remains widely used for LAA sizing, studies suggest that CT may offer superior accuracy. Yosefy et al. found that 2D TEE was non-inferior to CT in determining LAA area and volume, while 3D TEE represented a valuable alternative due to its high accuracy, lack of radiation exposure, and real-time bedside applicability [14]. Despite its lower temporal resolution, CECT provides a broader field of view, facilitating comprehensive anatomical assessment and procedural planning [14].

Recent advances in CECT post-processing have further improved its predictive capabilities for device sizing and implantation angles, showing excellent correlation with intra-procedural 3D TEE.

### 2.2. Intra-Procedural Imaging: Guidance During the Intervention

**Figure 3:**
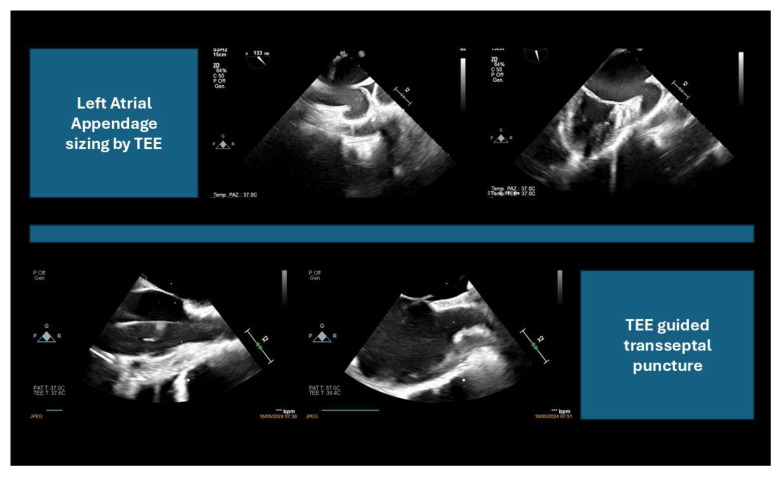
Left atrial appendage transesophageal echocardiography imaging: sizing and transseptal puncture.

**Figure 4:**
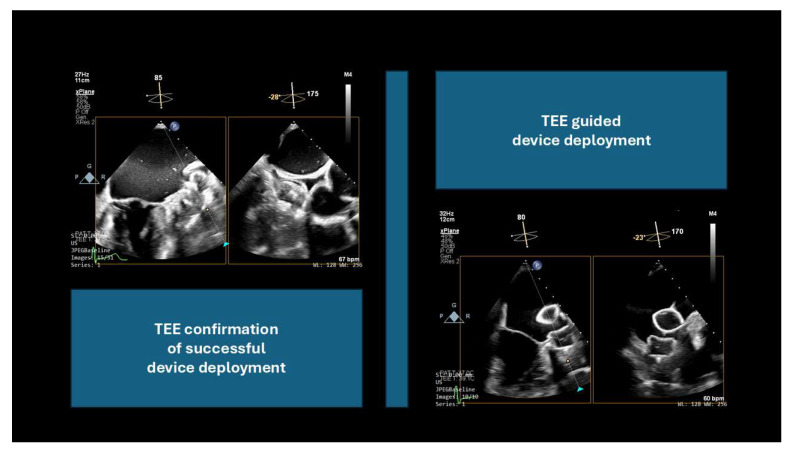
Left atrial appendage transesophageal echocardiography imaging: device deployment and positioning confirmation.

TEE and ICE, in conjunction with fluoroscopy, are the primary imaging modalities used to guide LAAC [15] (Figure 3 and Figure 4).

Both techniques provide real-time visualization of the left atrial anatomy; however, their 2D nature complicates the comprehensive assessment of LAA morphology. The 3D TEE option has significantly enhanced intra-procedural imaging by offering anatomically realistic “surgical views”, improving procedural guidance, peri-procedural communication, and post-deployment assessment. Zhou et al. demonstrated that 3D TEE provides greater accuracy than 2D TEE when evaluating LAA morphology, measuring the landing zone, depth, and ostium dimensions. Following device deployment, 3D TEE enables the single-view visualization of peri-device leaks, aiding in immediate procedural adjustments [16]. Additionally, Salzman et al. found that 3D-TEE-derived area and perimeter-based measurements closely correlate with the actual device size selected, highlighting its superior reproducibility compared with 2D TEE [17]. Moreover, real-time 3D TEE has proven to be effective in assessing key anatomical parameters—including the number of LAA lobes, ostium area, maximal and minimal diameters, and LAA depth—with accuracy comparable to CECT and static 3D TEE, as reported by Yosefy et al. [14].

Growing interest in intracardiac echocardiography (ICE) has led to its increasing adoption for LAAC guidance, particularly as it enables a single-operator approach, eliminating the need for general anesthesia and esophageal intubation with TEE. According to Garg et al., ICE enhances procedural efficiency while reducing patient discomfort, making it a valuable alternative. However, ICE-guided LAAC is technically more demanding, requiring the probe to be positioned within the left atrium for optimal imaging of the LAA, which necessitates greater operator expertise [18]. It is important to note, however, that the single-operator approach, while advantageous in certain settings, may not always be ideal, particularly when deploying nitinol-based occluders such as the WATCHMAN or AMULET devices. These implants can demonstrate variable behavior during release, and their successful deployment often benefits from a coordinated two-operator technique. In this scenario, one operator manipulates the delivery sheath (typically with a counterclockwise torque) while the second manages the device deployment under direct imaging guidance. This collaborative approach can improve precision and control, especially in complex anatomies or when device positioning proves challenging.

### 2.3. Post-Procedural Imaging: Evaluating Success and Follow-Up

Imaging plays a critical role not only in guiding device deployment and confirming adequate compression and sealing before release but also in the early detection of complications such as pericardial tamponade, air embolism, and thromboembolism. Transthoracic echocardiography (TTE) may be useful before discharge to monitor for immediate post-procedural complications.

For long-term evaluation, it is recommended to wait at least 45 days post-implantation before assessing the device as this represents the typical timeframe for endothelialization. CECT and TEE are the primary imaging modalities for evaluating device position, stability, and potential embolization. Both techniques provide high-resolution 3D imaging, enabling the precise visualization of the device within the LAA. This detailed assessment ensures secure positioning and effective sealing, ultimately reducing the risk of stroke and thromboembolism [19].

## 3. Current Imaging Techniques: Strengths and Limitations

### TEE Versus CT: Technical Challenges and Practical Considerations

The limitations of TEE, primarily due to its invasiveness, are increasingly recognized, particularly in elderly patients. Absolute and relative contraindications include esophageal pathology, coagulopathies, and severe thrombocytopenia. Additionally, anatomical variabilities in the LAA, the ostium’s position relative to the TEE probe, and individual heart positioning within the thoracic cavity can all impact optimal LAA visualization. Hemodynamic fluctuations and LAA contractile cycles may also lead to an underestimation of the ostium size. Finally, the lack of consistent anatomical landmarks makes it challenging to precisely determine the optimal landing zone for the occlusion device.

CECT represents a non-invasive alternative with a high spatial resolution, enabling multiplanar and 3D reconstructions of the LAA and surrounding structures. Dedicated CECT software allows for device implantation simulation, access route planning, and overlay/fusion imaging during the procedure. Compared with 2D TEE, pre-procedural CECT planning provides more accurate device sizing, reducing the procedural time, contrast usage, and potential complications. Additionally, optimized transseptal puncture site planning facilitates the coaxial alignment of delivery sheaths and devices within the LAA, while fluoroscopic simulation determines the optimal intra-procedural C-arm projection based on the CECT dataset [20].

Several studies have demonstrated the benefits of 3D modeling from CECT for LAAC procedural planning. One study reported reductions in the number of implanted prostheses, incidence of leaks, fluoroscopy time, and radiation dose compared with conventional imaging [20,21]. Other studies have shown fewer device and guide catheter changes and shorter procedural times with 3D modeling compared with TEE, along with a significant reduction in radiation exposure [22,23]. A meta-analysis of four studies confirmed that 3D CECT significantly reduces the incidence of periprosthetic leaks [24]. Compared with TEE, CT-derived 3D modeling for device planning has demonstrated the following [21,25]:

- Reduced total fluoroscopy time;
- Lower risk of periprosthetic leaks;
- Fewer devices used and shorter procedural times;
- Comparable device implantation success rates.

However, CECT is limited by contrast requirements, making it unsuitable for patients with severe renal impairment or contrast allergies. Although radiation exposure remains a concern, modern scanners and acquisition protocols have reduced the effective dose to 1–2 mSv [26].

CECT is highly sensitive when detecting LAA thrombi but false positives can occur if the image acquisition settings are not optimized. A delayed acquisition phase significantly improves the positive predictive value and specificity of cardiac CT (>95%) [27,28].

Additional limitations include heart rate control as a stable and slower rate is essential for optimal image quality. In atrial fibrillation (AF) patients, rate control medication may be required. Contrast tracking for synchronizing image acquisition with peak cardiac enhancement is also challenging as errors can result in low-contrast images. Motion artifacts remain a concern, although faster scanners and proper patient preparation help mitigate this issue [29]. Lastly, a higher body mass index (BMI) can increase image noise and artifacts, making CT imaging more challenging [30].

The combination of fluoroscopy and TEE remains the predominant imaging approach in many catheterization laboratories due to its established efficacy. However, intracardiac echocardiography (ICE) is gaining interest as an alternative, particularly for minimally invasive, single-operator procedures.

A key advantage of ICE-guided LAAC is that it can be performed under mild sedation, unlike TEE, which often requires general anesthesia or deep sedation. This translates into the following [31]:

1. Reduced anesthesia-related risks (avoiding endotracheal intubation);
2. Shorter procedural times (from femoral access to completion);
3. Faster patient recovery;
4. Decreased number of personnel in the operating room (eliminating the need for an anesthesiologist and imaging cardiologist).

Additionally, ICE reduces radiation exposure for medical staff, especially imaging cardiologists, who are frequently positioned near the X-ray source. This simplifies procedural workflow by involving fewer medical specialties and potentially improves scheduling flexibility.

TEE, although widely used, has been associated with esophageal injuries, detectable via esophagogastroduodenoscopy. Although these are often mild or asymptomatic, their clinical relevance should not be overlooked [32].

Despite its benefits, ICE-guided LAAC poses challenges, particularly for less experienced operators, as it requires them to perform both the procedure and imaging. This may explain the higher risk of pericardial effusion seen early in the learning curve. Notably, a study found that 82% of operators had performed fewer than 10 ICE-guided procedures, and an increased procedural volume correlated with a lower risk of complications. Conversely, a meta-analysis of observational studies where operators had substantial ICE experience found no significant differences in pericardial effusion or tamponade risk compared with TEE-guided procedures [33].

No major differences have been observed in procedural or fluoroscopy times between ICE and TEE. However, ICE guidance appears to significantly reduce the total time spent in the procedure room. Studies have reported comparable procedural efficacy and safety between the two modalities, with high procedural success rates and a low incidence of major adverse events [34].

Concerns regarding ICE’s ability to rule out LAA thrombi appear unfounded as studies indicate that ICE imaging has comparable accuracy to TEE in thrombus detection [35].

Despite these advantages, ICE adoption in routine clinical practice has been relatively slow, largely due to the following:

1. A steep learning curve, particularly for interventional cardiologists unfamiliar with left atrial imaging;
2. High costs of ICE catheters and equipment;
3. Uncertainty regarding device release criteria as TEE has traditionally been the gold standard for confirming device positioning.

CECT has emerged as a valuable tool for pre-procedural planning, allowing for precise device sizing, shorter procedural times, and reduced risk of complications associated with excessive probe manipulation. Dedicated software enables virtual ICE probe placement simulations, enhancing procedural precision and operator confidence [21]. See Table 1 for more details.

## 4. Innovation and Emerging Development

### 4.1. Three-Dimensional and Four-Dimensional Ultrasound

The accurate and reliable determination of LAA morphology is essential for percutaneous occlusion procedures [36,37].

Traditionally, 2D echocardiography, either TEE or ICE, in combination with contrast fluoroscopy has been the imaging modality of choice for guiding device selection and deployment and confirming appendage sealing.

However, 2D echocardiography provides images along a single cross-sectional plane, making a comprehensive assessment of the complex and highly variable LAA anatomy particularly challenging [38].

Three-dimensional (3D) echocardiography offers the direct visualization of LAA anatomy in multiplane and multislice modes with high reproducibility and without the need for geometric assumptions.

The 3D TEE option has been shown to provide significantly larger measurements of the LAA ostium and landing zone diameters compared with 2D TEE, with improved accuracy and reduced inter- and intra-observer variabilities [39].

Additionally, 3D TEE measurements are closer to those obtained via computed tomography (CT) and may even be more precise [14,40,41].

Enhanced accuracy aids the selection of appropriately sized devices, reducing procedural complications, radiation exposure, and procedural times [42].

Although TEE is a standard imaging modality, its use requires general anesthesia and is associated with complications, including esophageal laceration, perforation, and hemorrhage [18,43,44,45,46,47]. Moreover, TEE requires a dedicated operator to manipulate the probe during image acquisition [48].

Conversely, ICE only requires conscious sedation, without the need for general anesthesia, and can be handled by a single operator [18,43,48,49].

Recent studies have demonstrated that 2D-ICE is as safe and effective as TEE in guiding LAAC procedures, with the added benefit of shorter procedural times [50,51,52,53,54].

However, its lack of multiplane imaging capabilities can limit anatomical assessment.

The advent of real-time 3D-ICE, also referred to as four-dimensional (4D) ICE, addresses these limitations, resulting in accurate volumetric measurements, and real-time spatial orientation [50,55].

When combined with color Doppler imaging, 4D-ICE allows for a precise evaluation of peri-device flow and post-deployment leakage, improving procedural outcomes [56].

In a recent real-world study, the NUVISION 4D-ICE catheter provided superior guidance during device deployment compared with 2D-ICE and showed greater consistency in LAA sizing with pre-procedural TEE. Overall, the technical and procedural success rate of 4D-ICE-guided LAAC was 100%, with fewer patients requiring device recapture to achieve optimal placement and sealing [57].

In conclusion, robust evidence supports the superiority of 3D TEE over 2D TEE.

Although 2D-ICE is limited by its lack of multiplane imaging, the introduction of 4D-ICE is a game-changer. It holds the potential to replace TEE as the primary imaging modality for LAAC procedures, especially in patients with comorbidities like renal dysfunction where minimizing contrast use is desirable [58,59].

### 4.2. Three-Dimensional Printing

Three-dimensional (3D) printing refers to the fabrication of patient-specific cardiac anatomic replicas based on volumetric imaging datasets obtained by echocardiography or CT imaging. It allows for visualization and enhanced anatomic and hemodynamic understanding, improving procedural planning and allowing interventional simulation [60].

Data from the PROTECT AF trial show that 1.8 devices were used on average per procedure [61].

Improper device sizing and intra-procedural device or catheter exchanges increase the risk of complications, which include LAA perforation, pericardial effusion or tamponade, device embolization or migration, air or clot embolism, and compression of the left circumflex artery [62,63,64,65,66].

To address this problem, pre-procedural planning aided by the 3DP of LAA models has gained prominence in the literature [67].

Early proof-of-concept studies demonstrated the feasibility of implementing 3DP in LAAC procedures to predict the appropriate device size for implantation. These initial reports used a variety of devices, including WATCHMAN, AMULET, WaveCrest, and highlighted the potential of 3DP to enhance procedural planning [64,68,69,70].

Building on these findings, subsequent studies expanded patient cohorts to systematically compare the accuracy of sizing predictions between 3DP-based approaches and conventional imaging modalities such as 2D/3D TEE and CT imaging. The results from five prospective studies demonstrated that 3DP-based strategies outperformed imaging-based approaches, achieving accurate device size predictions in up to 100% of cases [62,71,72,73,74].

This improvement resulted in a reduced number of devices used, decreased the fluoroscopic time, and demonstrated a marked reduction in peri-device leak rates [21,75].

In summary, there is some evidence supporting the use of 3DP LAA models as a complement to conventional imaging-based sizing strategies. However, the available data remain limited, and incorporating 3DP into the LAAC workflow adds to the pre-procedural planning time.

### 4.3. Fusion Imaging

Fusion imaging has recently emerged as an innovative tool employed to guide interventional procedures [76,77].

This approach integrates fluoroscopy with other imaging modalities such as TEE or CT imaging to overcome the limitations of individual techniques.

CT imaging is increasingly becoming the standard in pre-procedural planning for LAAC.

Compared with TEE, CT provides better visualization of the LAA anatomy, allowing accurate device sizing and placement, which reduces the likelihood of under-sizing, minimizes the number of devices required, and shortens procedural times [23,61].

Dedicated fusion imaging software uses fluoroscopic landmarks to overlay the CT-derived LAA anatomy onto live fluoroscopic images in real time. This integration allows a significant reduction in the contrast media volume needed, shortens procedural and fluoroscopy times with a trend toward improved device deployment success rates, and facilitates complete sealing [78].

Although fluoroscopy excels at visualizing catheter and device positions, it offers limited anatomical detail, providing only 2D images of the lumen. Conversely, TEE delivers high-resolution imaging of cardiac structures, including the LAA walls, with the added benefit of 3D visualization.

Fusion imaging combines these complementary strengths using specialized software platforms such as the EchoNavigator system (Philips Healthcare, Best, The Netherlands) and TrueFusion (Siemens Healthineers, Erlangen, Germany). These systems synchronize real-time echocardiographic and fluoroscopic imaging using calibration algorithms that track TEE probe movements using fluoroscopy [79].

**Figure 5:**
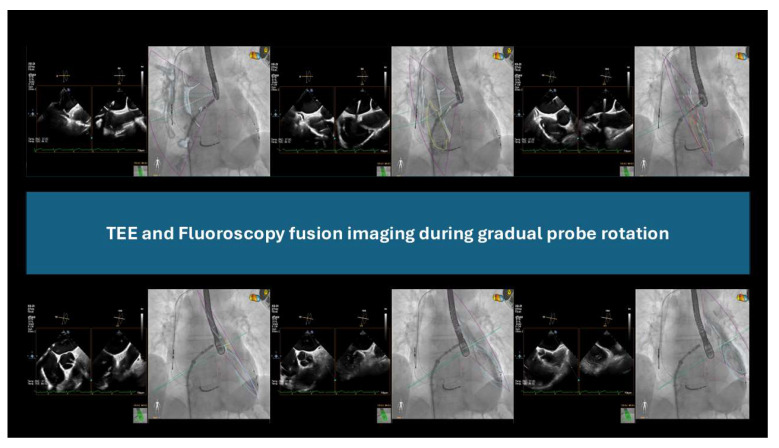
Transesophageal echocardiography and fluoroscopy fusion imaging.

The integration of TEE and fluoroscopy has recently been applied to LAAC procedures involving both AMULET and WATCHMAN devices. This technology reduces radiation exposure, shortens procedure durations, facilitates transseptal puncture, and minimizes the amount of contrast agent required. Remarkably, fusion imaging may even overcome the need for contrast injection [80,81,82,83,84,85] (Figure 5).

In summary, fusion imaging combines the strengths of different imaging modalities and shows potential for enhancing procedural efficiency. However, evidence regarding its impact on clinically meaningful procedural results remains limited and its use adds procedural complexity.

### 4.4. AI and Deep Learning

Artificial intelligence (AI) and deep learning are increasingly being integrated into percutaneous structural heart disease interventions. An example is the combination of AI with advanced 3D printing techniques, enabling the creation of patient-specific anatomical replicas. These replicas enhance procedural training and contribute to better outcomes by simulating realistic anatomical scenarios, potentially improving outcomes and operator confidence [86].

In the context of pre-procedural computed tomography (CT) analyses, AI-based automation offers significant advantages, including time efficiency, reduced inter-observer variability, and increased standardization.

For LAAC, a fully automated AI-based CT analysis can be achieved using supervised deep learning algorithms trained on annotated datasets derived from manual expert segmentation. These models can accurately detect critical key anatomical landmarks, including the ostium, landing zone, mitral valve annulus, and fossa ovalis, as well as perform reliable segmentation of the LA and LAA. Validation studies have demonstrated that the variability between AI-predicted and manual assessments is comparable to inter-operator variability, with the added benefit of significantly reduced analysis time [87].

Patient-specific computational modeling is another promising innovation, providing insights into the interaction between the device and the patient’s anatomy, which may enhance the selection of the LAA closure device size and implant position [88].

A notable example of this technology is FEops HEARTguide, a platform that uses AI-enhanced anatomical analyses combined with finite element modeling to create dynamic simulations of device deployment based on patient CT data. This tool predicts critical aspects such as device deformation, wall apposition, and the likelihood of peri-device leaks.

By integrating AI-driven anatomical analyses with computer simulations, this platform predicts the compatibility of each device with the particular patient’s auricle anatomy.

The clinical value of FEops HEARTguide has been demonstrated in real-world settings, showing high concordance between simulated and actual outcomes.

In the prospective, multicenter, randomized PREDICT-LAA trial, the use of FEops HEARTguide significantly improved procedural efficiency and showed a trend toward better outcomes [89].

In clinical practice, the platform allows for a correct device change in nearly one-third of patients compared with the standard of care [90], suggesting a meaningful impact on procedural planning and success.

The application of AI and deep learning technologies in pre-procedural CT analyses for LAAC enables a more automated, standardized, and patient-focused approach with the potential to improve procedural success. Supervised, semi-automated AI-based CT analyses will become a standard in clinical practice, offering faster and more efficient reporting. As these algorithms continue to evolve with the integration of additional data in the coming years, we look forward to the advancements and outcomes they will help us to achieve.

## 5. Conclusions

The evolution of imaging techniques for left atrial appendage closure (LAAC) has significantly enhanced procedural planning, execution, and outcomes.

Traditional imaging modalities such as transesophageal echocardiography (TEE) and intracardiac echocardiography (ICE) have long been the cornerstone of procedural guidance, yet they present limitations in terms of visualization, invasiveness, and required expertise. Recent advancements in three-dimensional (3D) and four-dimensional (4D) ultrasound, fusion imaging, 3D printing, and artificial intelligence (AI) have introduced groundbreaking solutions that address these challenges.

The introduction of 3D TEE and 4D-ICE has revolutionized real-time imaging by providing volumetric and multiplanar reconstructions of the left atrial appendage (LAA). These modalities enable more precise device sizing and positioning, reducing procedural complications and the need for multiple device deployments. Notably, 4D-ICE has emerged as a promising alternative to TEE, eliminating the need for general anesthesia and enhancing procedural efficiency while maintaining diagnostic accuracy.

Three-dimensional printing has further refined pre-procedural planning by allowing patient-specific modeling of the LAA. This technology has demonstrated its potential in optimizing device selection, reducing procedural time, and minimizing complications such as peri-device leaks. However, its widespread adoption remains limited due to the additional time and resources required for model fabrication.

Fusion imaging, which integrates fluoroscopy with either contrast-enhanced computed tomography (CECT) or echocardiographic imaging, has addressed the limitations of standalone imaging techniques by improving anatomical visualization and reducing contrast media use and radiation exposure.

AI and deep learning are being increasingly adopted to optimize LAAC procedures. AI-powered CECT analyses have shown promising results in automating pre-procedural planning, improving standardization, and reducing operator variability. Computational modeling and AI-driven simulations have demonstrated significant potential in enhancing device selection and predicting procedural success.

Future studies should focus on validating these advancements through large-scale, multicenter trials to establish their definitive role in clinical practice.

## Figures and Tables

**Table 1:** Advantages and disadvantages of imaging techniques used for LAAO. LAA: left atrial appendage; LAAO: left atrial appendage occlusion; 2D: two-dimensional; 3D: three-dimensional.

Method	Advantages	Disadvantages
Transesophageal Echocardiography (TEE)	Widely used and available. High-resolution imaging of cardiac structures, including the LAA walls, with 3D visualization. Good accuracy when evaluating LAA morphology, measuring the landing zone, depth, and ostium dimensions, and aiding in the visualization of peri-device leaks. The 3D TEE option provides significantly larger and more accurate measurements of the LAA ostium and landing zone diameters compared with 2D TEE. High temporal resolution, useful when monitoring and guiding periprocedural implantation.	Invasive, particularly for elderly patients. Contraindications include esophageal pathology, coagulopathies, and severe thrombocytopenia. Anatomical LAA and heart variants can impact optimal LAA visualization. Hemodynamic fluctuations and LAA contractile cycles may lead to an underestimation of the ostium size. May require general anesthesia and associated complications, including esophageal laceration, perforation, and hemorrhage. Requires a dedicated operator to manipulate the probe during image acquisition.
Contrast-Enhanced Computed Tomography (CECT)	Non-invasive alternative. High spatial resolution, enabling multiplanar and 3D reconstructions of the LAA and surrounding structures. Highly sensitive at detecting LAA thrombi. Dedicated software allows for device implantation simulation, access route planning, and overlay/fusion imaging. Pre-procedural CECT planning provides more accurate device sizing, reducing procedural time, contrast usage, and potential complications. Optimized transseptal puncture site planning facilitates coaxial alignment and fluoroscopic simulation determines the optimal intra-procedural C-arm projection. Three-dimensional modeling from CECT has demonstrated benefits, including reductions in the number of implanted prostheses, incidence of leaks, fluoroscopy time, and radiation dose.	Radiation exposure is a concern. Contrast requirements are unsuitable for patients with severe kidney impairment or allergies. False positives for LAA thrombi if image acquisition settings are not optimized. Heart beat synchronization, which may necessitate medication, or can result in motion artifacts in AF patients. Motion artifacts remain a concern (e.g., breathing during acquisition). Higher body mass index (BMI) can increase image noise and artifacts.
Intracardiac Echocardiography (ICE)	Feasible for a single-operator approach. Eliminates the need for general anesthesia and esophageal intubation while reducing patient discomfort and facilitates faster patient recovery. Enhances procedural efficiency and shortens procedural times. Reduces radiation exposure for medical staff. The 2D-ICE option is as safe and effective as TEE. The 4D-ICE option provide accurate volumetric measurements and real-time spatial orientation. Comparable accuracy to TEE in thrombus detection.	Technically more demanding, requiring greater operator expertise. Challenges for less experienced operators, with a higher risk of pericardial effusion early in the learning curve. Steep learning curve. High costs of ICE catheters and equipment. Uncertainty regarding device release criteria.

## Data Availability

Not applicable.

## Supplementary Materials

The following supporting information can be downloaded at: https://www.mdpi.com/article/10.3390/jcm14103607/s1, Supplementary Materials; Systematic Comparison of Guidelines.
