# Supplementary material for: Optimizing Imaging Techniques for Left Atrial Appendage Closure: Insights and Emerging Directions
Source: J Clin Med. 2025 May 21;14(10):3607. doi: 10.3390/jcm14103607 (PMC12112684; doi:10.3390/jcm14103607)
Supplement: Supplementary file 1 [file jcm-14-03607-s001.zip › jcm-3580293-supplementary.pdf]

Systematic Comparison of Guidelines

Current international guidelines converge on the central role of multimodality imaging in all phases of left atrial appendage closure (LAAC), although differences exist in their specific recommendations and assigned levels of evidence. The 2024 ESC Guidelines on atrial fibrillation management recommend pre-procedural imaging with contrast-enhanced cardiac CT (CECT) or transesophageal echocardiography (TEE) to assess LAA morphology and exclude thrombus (Class IIa, Level of Evidence B), and post-procedural imaging for device surveillance is similarly endorsed (Class IIa, Level of Evidence C). The 2023 AHA/ACC guideline also supports TEE or CECT for pre-procedural planning and thrombus exclusion (Class IIa, Level B), and highlights intracardiac echocardiography (ICE) as a reasonable alternative to TEE during the procedure, particularly in patients who cannot tolerate general anesthesia (Class IIb, Level B). The Japanese Circulation Society (JCS) guidelines primarily emphasize the use of TEE for both diagnostic and procedural purposes, with a Class IIa recommendation for its use in guiding LAAC, while recognizing CECT as an adjunctive modality in anatomically complex cases or when TEE is contraindicated. Across all documents, the choice of imaging modality is advised to be individualized based on patient characteristics, procedural complexity, and institutional expertise (generally Class I or IIa recommendations, depending on the context). It should be noted that fusion imaging and artificial intelligence have not yet been comprehensively addressed within current guidelines and remain emerging tools whose clinical implementation is mainly supported by early studies and expert consensus.

**Imaging Modality**	**ESC 2024**	**AHA/ACC 2023**	**JCS**	**Comments**
Transesophageal Echocardiography (TEE)	Class IIa, Level B (pre- and post-procedural)	Class IIa, Level B (pre- and intra-procedural)	Class IIa, Level B	First-line imaging tool across all guidelines; essential for thrombus exclusion and guidance
Intracardiac Echocardiography (ICE)	Not explicitly graded; mentioned as an alternative	Class IIb, Level B	Not routinely addressed	AHA/ACC supports ICE as an alternative to TEE; limited references in ESC; not yet endorsed by JCS
Contrast-Enhanced Cardiac CT (CECT)	Class IIa, Level B (pre-procedural)	Class IIa, Level B	Mentioned; no formal classification	Increasingly used for anatomical assessment and procedural planning
Fusion Imaging	Mentioned; not formally graded	Not mentioned	Not mentioned	Emerging modality; improves anatomical integration but not yet included in formal guidelines
AI-Enhanced Analysis/Computational Modeling	Mentioned; not formally graded	Not mentioned	Not mentioned	Experimental approach; promising but still outside current guideline frameworks

References

1. Van Gelder, I.C.; Rienstra, M.; Bunting, K.V.; Casado-Arroyo, R.; Caso, V.; Crijns, H.J.G.M.; De Potter, T.J.R.; Dwight, J.; Guasti, L.; Hanke, T.; et al. 2024 ESC Guidelines for the management of atrial fibrillation developed in collaboration with the European Association for Cardio-Thoracic Surgery (EACTS). *Eur. Heart J.* **2024**, *45*, 3314–3414. https://doi.org/10.1093/eurheartj/ehae176. PMID: 39210723.
2. Joglar, J. A.; Chung, M. K.; Armbruster, A. L.; Benjamin, E. J.; Chyou, J. Y.; Cronin, E. M.; Deswal, A.; Eckhardt, L. L.; Goldberger, Z. D.; Gopinathannair, R. *et al* 2023 ACC/AHA/ACCP/HRS Guideline for the Diagnosis and Management of Atrial Fibrillation: A Report of the American College of Cardiology/American Heart Association Joint Committee on Clinical Practice Guidelines. *Circulation* **2024**, *149*, e1–e156. https://doi.org/10.1161/CIR.0000000000001193.
3. Chao, T. F.; Joung, B.; Takahashi, Y.; Lim, T. W.; Choi, E. K.; Chan, Y. H.; Guo, Y.; Sriratanasathavorn, C.; Oh, S.; Okumura, K. *et al.* 2021 Focused Update Consensus Guidelines of the Asia Pacific Heart Rhythm Society on Stroke Prevention in Atrial Fibrillation: Executive Summary. *Thromb. Haemost.* **2022**, *122*, 20–47. https://doi.org/10.1055/s-0041-1739411.
